# Combined effect of left ventricular ejection fraction and obesity on sedentary behavior in patients with coronary artery disease

**DOI:** 10.1097/MD.0000000000035839

**Published:** 2023-11-10

**Authors:** Mi Hwa Won, JaeLan Shim

**Affiliations:** a Department of Nursing, Wonkwang University, Iksan, South Korea; b College of Nursing, Dongguk University, Gyeongju, South Korea.

**Keywords:** cardiac function, coronary artery disease, obesity, sedentary behavior

## Abstract

Sedentary behavior has been associated with poor adherence to treatment in patients with coronary artery disease. Low left ventricular ejection fraction and obesity have been separately linked to increased sedentary behavior in patients with coronary artery disease. However, the combined effect of low left ventricular ejection fraction and obesity on sedentary behavior in patients with coronary artery disease has not been thoroughly investigated. Therefore, this study aimed to examine the combined influence of left ventricular ejection fraction and obesity on sedentary behavior in patients with coronary artery disease. This descriptive cross-sectional study enrolled 200 inpatients aged ≥ 20 years who were diagnosed with coronary artery disease at a tertiary hospital in Korea between March and August 2022. Data were collected using structured questionnaires, and multivariate logistic regression analysis was performed to determine the combined effect of left ventricular ejection fraction and obesity on sedentary behavior in patients with coronary artery disease. Among the 111 patients with sedentary behavior, 38 (34.2%) had both low left ventricular ejection fraction and obesity, whereas only 11 (12.4%) of the 89 patients without sedentary behavior had both low left ventricular ejection fraction and obesity. In multivariate logistic regression analysis, patients with coronary artery disease who had both low left ventricular ejection fraction and obesity had the highest risk of sedentary behavior compared to those without either low left ventricular ejection fraction or obesity (odds ratio = 13.98, 95% confidence interval = 5.19–37.69, *P <* .001). The co-existence of low left ventricular ejection fraction and obesity in patients with coronary artery disease may be associated with sedentary behavior. Therefore, evaluating both left ventricular ejection fraction and obesity when assessing sedentary behavior in patients with coronary artery disease may be valuable in implementing patient-centered approaches for the secondary prevention and management of sedentary behavior in patients with coronary artery disease. However, further prospective cohort studies with larger sample sizes are required to establish causal relationships and explore interventions to mitigate sedentary behavior in this population.

## 1. Introduction

Coronary artery disease (CAD) is a prevalent global health issue closely associated with chronic diseases, such as hypertension, diabetes, and atherosclerosis.^[1]^ As the population ages, the prevalence of these risk factors is expected to rise.^[2]^ CAD can lead to severe complications, including stroke, heart failure, and chronic kidney disease, significantly impacting individuals and the overall economy.^[3]^ Therefore, implementing appropriate management strategies for CAD, including pharmacological therapy and lifestyle modifications, is crucial.^[4]^ Moreover, identifying modifiable risk factors for CAD, such as sedentary behavior, is critical for developing effective secondary prevention strategies for patients with CAD.

Sedentary behavior is any waking behavior characterized by sitting, resting, or lying down with low energy expenditure.^[5]^ It is widely recognized as a major modifiable risk factor for cardiovascular disease mortality.^[5]^ In addition to its association with cardiovascular diseases, sedentary behavior has been linked to various negative health outcomes, including an increased risk of recurrent events and mortality in patients with CAD.^[6]^ Recent studies have provided strong evidence of the association between prolonged sedentary behavior and CAD regarding development and recurrence.^[7,8]^ Additionally, population-based studies have demonstrated that sedentary behavior is associated with higher CAD morbidity and mortality rates as well as increased susceptibility to CAD risk factors, such as insulin resistance.^[6]^ Moreover, a previous study reported that each additional hour of sedentary behavior increased the likelihood of coronary artery calcium deposition by 12%, thereby increasing CAD incidence.^[9]^ In this context, a prospective observational study showed that patients with myocardial infarction and a high sedentary behavior had a 62% higher mortality rate than those with a low sedentary behavior.^[10]^

Despite the negative health outcomes associated with sedentary behavior in patients with CAD, these patients still engage in high levels of sedentary behavior.^[11]^ A longitudinal study showed that patients with CAD spend 10.4 h/d being sedentary, which is higher than that of a healthy population.^[12]^ Additionally, an observational cohort study reported high sedentary behavior (9.7 ± 2.0 h/d) in patients with acute coronary syndrome at 28 days post-discharge.^[13]^ Therefore, identifying the factors that affect sedentary behavior is necessary to continuously reduce sedentary behavior that influences the disease progression and prognosis in patients with CAD.

Left ventricular ejection fraction (LVEF) is a widely used parameter to assess the pumping efficiency of the heart.^[14]^ It serves as an essential indicator for evaluating the performance of the left ventricle, which plays a crucial role in pumping oxygen-rich blood throughout the body.^[14]^ In patients with CAD, LVEF is an important indicator of cardiac health and can provide insights into disease severity and prognosis.^[4]^ Patients with low LVEF can experience symptoms, such as fatigue and shortness of breath, because the heart cannot pump the right amount of blood to meet the body’s needs, contributing to sedentary behavior.^[15]^ Several studies have suggested a potential correlation between sedentary behavior and LVEF decline.^[13,15]^ A previous study reported that patients with CAD with <40% LVEF are more likely to have high sedentary behavior than those with LVEF > 40%.^[13]^ Finally, a clear relationship between low LVEF and sedentary behavior in patients with CAD may help counter sedentary behavior and improve cardiovascular health by encouraging regular physical activity within an individual’s cardiac limitations.

Obesity is a well-established risk factor for CAD and is recognized as the primary cause of recurrent CAD events in patients with CAD.^[16]^ It can contribute to the development of various conditions, including hypertension, dyslipidemia, and insulin resistance.^[4]^ Collectively, these factors increase the risk of recurrent events in individuals with obesity who have underlying CAD.^[16]^ The prevalence of obesity among patients with coronary syndrome is substantial in Europe. Furthermore, many patients participating in cardiac rehabilitation programs have been observed as obese.^[17]^

Patients with CAD who exhibit high levels of sedentary behavior are more prone to weight gain and obesity. This is primarily because of prolonged periods of sitting or inactivity, which can lead to decreased energy expenditure. Additionally, this disrupts the balance between calorie intake and expenditure.^[18]^ An interventional study involving patients with CAD who participated in a 12-week exercise-based cardiac rehabilitation program showed that participants with obesity exhibited higher levels of sedentary behavior than those with normal weight.^[19]^

Although previous studies have suggested a connection between sedentary behavior and CAD, as well as their association with LVEF and obesity, knowledge regarding the simultaneous relationship between LVEF, obesity, and sedentary behavior in patients with CAD is limited. Therefore, this study primarily aimed to investigate the combined effect of LVEF and obesity on sedentary behavior among patients with CAD. We hypothesized that the concurrent occurrence of LVEF and obesity would increase the risk of sedentary behavior in patients with CAD.

## 2. Materials and methods

### 2.1. Study design and sample

This descriptive observational study included patients who were diagnosed with CAD and hospitalized in the cardiology ward of a tertiary hospital in South Korea. A convenience sampling method was employed to select the enrolled participants between March and August 2022. The inclusion criteria were as follows: age ≥ 20 years; confirmed diagnosis of CAD; ability to communicate and understand Korean; and willingness to participate in the study. The following were the exclusion criteria: individuals diagnosed with stroke or those with memory impairment, mental disorder, end-stage renal disease, or progressive cancer.

The required sample size was determined using G*Power 3.1.9.2. A total of 193 patients were required to achieve a power of 95% at a significance level of 0.05, with a two-tailed logistic regression analysis and an odds ratio of 1.95. Considering the potential dropouts, 200 patients with CAD were recruited for this study. Ultimately, data analysis was conducted on 200 patients.

### 2.2. Ethical considerations

This study was approved by the Institutional Review Board (number: WKIRB-202202-SB-011). Written informed consent was obtained from all the patients who agreed to participate after clearly explaining the study’s objectives and procedure. To adhere to the General Data Protection Regulation guidelines, strict measures were taken to ensure anonymity and confidentiality of the participants’ information. The completion of the structured questionnaire by patients was voluntary. Additionally, to safeguard the participants’ personal and sensitive information, efforts were made to minimize the inclusion of sentences about personal information.

### 2.3. Instruments

#### 2.3.1. Sociodemographic and clinical characteristics.

The patient characteristics were determined based on a comprehensive literature review.^[12,13,20]^ These characteristics included age, sex, educational level, living arrangement, employment status, current smoking, time since CAD diagnosis, Canadian Cardiovascular Society (CCS) angina grade, presence of chronic diseases (such as hypertension, diabetes mellitus, atrial fibrillation, and heart failure), prescribed cardiac medications (such as aspirin, clopidogrel, angiotensin-converting enzyme inhibitors, angiotensin II receptor blockers, beta-blockers, and lipid-lowering agents), clinical parameters (such as systolic blood pressure, diastolic blood pressure, and pulse rate), and laboratory parameters (such as total cholesterol, triglycerides, low-density lipoprotein cholesterol, high-density lipoprotein cholesterol, blood urea nitrogen, and creatinine).

The CCS angina grade is a classification system used to assess the severity of angina, which is described as chest pain or discomfort caused by reduced blood flow to the heart. The CCS angina grade consists of 4 grades as follows: Class I, no angina with routine activities and no limitation on physical exertion; Class II, angina that is slightly worse than during ordinary physical activity; Class III, angina that occurs when walking on level ground, at a slower pace, or while performing light activities; and class IV, angina at rest or with minimal physical activity.^[20]^

#### 2.3.2. Left ventricular ejection fraction.

The LVEF, measured via echocardiography, is a vital and objective marker for assessing disease severity. In this study, data recorded at admission, extracted from electronic medical records, were used for LVEF assessment. We have recently established a “normal” LVEF threshold as ≥55%, aligning with the cardiac dimension and function assessment’s normal range set by the British Society of Echocardiography,^[21]^ and drawing from recent research.^[14,22]^ Consequently, LVEF was categorized into “normal” (≥55%) and “low” (<55%) groups.

#### 2.3.3. Obesity.

Obesity was assessed using body mass index (BMI). The criteria for defining obesity were based on the Korean Society for Obesity guidelines, classifying individuals with a BMI of ≥ 25 kg/m^2^ as obese.^[22]^

#### 2.3.4. Sedentary behavior.

The evaluation of sedentary behavior in this study depended on a single item extracted from the widely validated Global Physical Activity Questionnaire. We used the validated Korean version of the Global Physical Activity Questionnaire. Participants were asked to report the time spent engaging in sedentary behavior, such as sitting or lying down, during various activities, including working at home, commuting, and socializing with friends. The assessment specifically excluded time spent sleeping. We utilized predetermined cutoff points (<8 and ≥8 hours) established by previous studies to categorize sedentary behavior.^[23,24]^ These studies demonstrated that engaging in sedentary behavior for ≥8 h/d is associated with an elevated risk of depressive symptoms, cardiovascular diseases, and overall mortality.

### 2.4. Data collection

Participants were recruited from a cardiology ward through a recruitment poster displayed on a bulletin board. Potential participants were identified by referring to hospital registries and reviewing their medical records. Two trained researchers conducted data collection. Written informed consent was obtained from each participant, and a self-reported questionnaire was administered to gather relevant information. The interview lasted approximately 10 to 20 minutes to ensure a thorough data collection. Clinical data were obtained from the electronic medical records.

### 2.5. Statistical analysis

The collected data were analyzed using IBM SPSS Statistics for Windows, version 26 (IBM Corp., Armonk, NY). The characteristics of patients with CAD with and without sedentary behavior were compared using the chi-squared test or *t* test, depending on the nature of the data. To further examine the association between sedentary behavior and independent variables while controlling for confounding variables, multivariate logistic regression analysis was conducted using the enter method. Participants were categorized into the following 4 groups to explore the combined influence of LVEF and obesity: normal LVEF without obesity, low LVEF alone, obesity alone, and low LVEF with obesity. The effect estimates are presented as odds ratios (ORs) with corresponding 95% confidence intervals (CIs). Statistical significance was set at *P* < .05.

## 3. Results

### 3.1. Sociodemographic and clinical characteristics of patients with CAD

The study included 200 patients with a mean age of 75.44 years (standard deviation [SD] = 6.51). Among these, 107 (53.5%) were aged ≥ 65 years, and 106 (53.0%) were male. By educational level, 54.5% of the patients had completed education beyond high school. Furthermore, 78.0% of the patients lived with their families, 50.0% were unemployed, and 79.0% were currently nonsmokers (Table 1).

**Table 1:** Sociodemographic characteristics of patients with CAD according to the presence of sedentary behavior (n = 200).

Characteristics	Total (n = 200), n (%)	Sedentary behavior, n (%)		χ^2^	*P*
		No (n = 89)	Yes (n = 111)		
Age (yr)					
<65	93 (46.5)	50 (54.9)	43 (39.4)	3.56	.040
≥65	107 (53.5)	41 (45.1)	66 (60.6)		
Sex					
Male	106 (53.0)	56 (62.9)	50 (45.0)	6.34	.015
Female	94 (47.0)	33 (37.1)	61 (55.0)		
Educational level					
≤middle school	91 (45.5)	34 (38.2)	57 (51.4)	3.44	.086
≥high school	109 (54.5)	55 (61.8)	54 (48.6)		
Living arrangement					
Living alone	44 (22.0)	20 (22.5)	24 (21.6)	0.02	.510
Living with family	156 (78.0)	69 (77.5)	87 (78.4)		
Employment					
Unemployed	100 (50.0)	47 (52.8)	53 (47.7)	0.51	.569
Employed	100 (50.0)	42 (47.2)	58 (52.3)		
Current smoking					
No	158 (79.0)	65 (73.0)	93 83.8)	4.39	.111
Yes	42 (21.0)	24 (27.0)	18 (16.2)		

CAD = coronary artery disease, SD = standard deviation.

Regarding the clinical characteristics, 107 (53.5%) patients had been diagnosed with CAD < 1 year ago, and 82 (41.0%) were classified into Class I according to the CCS criteria. The following proportion of patients had specific chronic conditions: hypertension (n = 132, 66.0%); diabetes mellitus (n = 73, 37.5%); atrial fibrillation (n = 29, 14.5%); and heart failure (n = 36, 18.0%). Most patients (n = 165, 82.5%) were taking clopidogrel. The mean systolic and diastolic blood pressures were 122.27 (SD = 14.45) and 71.69 (SD = 10.8) mm Hg, respectively.

### 3.2. Prevalence of sedentary behavior and comparison of the characteristics of patients with CAD with and without sedentary behavior

The prevalence of sedentary behavior among patients with CAD was 55.5% (n = 111). Tables 1 and 2 present the comparison of the characteristics of patients with CAD with and without sedentary behavior. Statistical analysis revealed significant differences in age (χ^2^ = 3.56, *P* = .040) and sex (χ^2^ = 6.34, *P* = .015) between the 2 groups. However, no significant differences were observed in other characteristics, such as educational level, living arrangement, employment status, current smoking, time since CAD diagnosis, CCS angina grade, comorbidities, prescribed medication usage, clinical parameters, or laboratory parameters among patients with CAD.

**Table 2:** Clinical characteristics of patients with CAD according to the presence of sedentary behavior (n = 200).

Characteristics	Total (n = 200), n (%) or mean (SD)	Sedentary behavior, n (%) or mean (SD)		χ^2^/*t*	*P*
		No (n = 89)	Yes (n = 111)		
Time since CAD diagnosis					
<1 yr	107 (53.5)	50 (56.2)	57 (51.4)	0.46	.599
≥1 yr	93 (46.5)	39 (43.8)	54 (48.6)		
CCS					
I	82 (41.0)	46 (51.7)	36 (32.5)	0.16	.732
II	75 (37.5)	25 (28.1)	50 (45.0)		
≥III	43 (21.5)	18 (20.2)	25 (22.5)		
Hypertension, yes	132 (66.0)	62 (69.7)	70 (63.1)	0.96	.369
Diabetes mellitus yes	75 (37.5)	28 (31.5)	47 (42.3)	2.49	.142
Atrial fibrillation, yes	29 (14.5)	14 (15.7)	15 (13.5)	0.20	.690
Heart failure, yes	36 (18.0)	12 (13.5)	24 (21.6)	2.21	.144
Aspirin, yes	138 (69.0)	62 (69.7)	76 (68.5)	0.03	.879
Clopidogrel, yes	165 (82.5)	73 (82.0)	92 (82.9)	0.02	.509
ACEI or ARB, yes	98 (49.0)	45 (50.6)	53 (47.7)	0.16	.776
Beta blocker, yes	79 (39.5)	34 (38.2)	45 (40.5)	0.11	.772
Lipid-lowering agents, yes	171 (85.5)	78 (87.6)	93 (83.8)	0.59	.546
Systolic BP (mm Hg)	122.27 (14.45)	121.53 (13.15)	122.86 (15.45)	−0.65	.517
Diastolic BP (mm Hg)	71.69 (10.81)	71.27 (10.43)	72.02 (11.13)	−0.48	.628
Pulse (beat/min)	69.84 (13.76)	69.30 (70.27)	13.59 (13.94)	−0.49	.623
Serum total cholesterol (mg/dL)	164.52 (54.84)	166.12 (54.67)	136.23 (55.19)	0.36	.713
Serum triglyceride (mg/dL)	138.98 (73.16)	138.80 (74.87)	139.13 (72.11)	−0.03	.975
Serum LDL-cholesterol (mg/dL)	104.65 (44.75)	107.39 (43.16)	102.45 (46.16)	0.77	.440
Serum HDL-cholesterol (mg/dL)	45.23 (25.46)	45.80 (17.53)	44.77 (30.44)	0.28	.776
Serum BUN (mg/dL)	22.33 (26.56)	23.12 (24.27)	21.69 (28.35)	0.78	.706
Serum creatine (mg/dL)	1.41 (3.43)	1.26 (2.23)	1.53 (4.16)	−0.53	.591

ACEI = angiotensin-converting enzyme inhibitor, ARB = angiotensin II receptor blocker, BP = blood pressure, BUN = blood urea nitrogen, CAD = coronary artery disease, CCS = Canadian Cardiovascular Society, HDL-cholesterol = high-density lipoprotein, LDL-cholesterol = low-density lipoprotein, SD = standard deviation.

### 3.3. Comparison of the LVEF and obesity prevalence in patients with CAD with and without sedentary behavior

Table 3 shows the prevalence of separate and combined categories of LVEF and obesity based on the presence of sedentary behavior. Among the patients, 49 (24.5%) had a low LVEF and were obese. Examination of the individual effect of LVEF or obesity showed that patients with sedentary behavior had a lower LVEF (χ^2^ = 17.03, *P* < .001) and a higher prevalence of obesity (χ^2^ = 9.50, *P* = .003) than those without sedentary behavior. Regarding the combined effect of LVEF and obesity, the proportion of patients with sedentary behavior with low LVEF and obesity (n = 38, 34.2%) was significantly higher than that of those without sedentary behavior (n = 11, 12.4%). In contrast, the proportion of patients with normal LVEF without obesity was 12.6% (n = 14) in the sedentary behavior group (χ^2^ = 24.52, *P* < .001).

**Table 3:** Comparison of LVEF and obesity in patients with CAD according to the presence of sedentary behavior (n = 200).

Characteristics	Total (n = 200), n (%)	Sedentary behavior, n (%)		χ^2^	*P*
		No (n = 89)	Yes (n = 111)		
Individual influence					
LVEF					
Normal	68 (34.0)	44 (49.4)	24 (21.6)	17.03	<.001
Low	132 (66.0)	45 (50.6)	87 (78.4)		
Obesity					
No	132 (66.0)	69 (77.5)	63 (56.8)	9.50	.003
Yes	68 (34.0)	20 (22.5)	48 (43.2)		
Combined influence					
Normal LVEF and without obesity	52 (26.0)	38 (42.7)	14 (12.6)	27.58	<.001
Low LVEF alone	83 (41.5)	34 (38.2)	49 (44.1)		
Obesity alone	16 (8.0)	6 (6.7)	10 (5.1)		
Low LVEF and obesity	49 (24.5)	11 (12.4)	38 (34.2)		

CAD = coronary artery disease, LVEF = left ventricular ejection fraction.

### 3.4. Effect of combined LVEF and obesity on sedentary behavior in patients with CAD

The association between the individual or combined effect of LVEF and obesity on sedentary behavior was examined using multivariate logistic regression analysis. The analysis was adjusted for age and sex, which were significant in the bivariate analysis (Table 4).

**Table 4:** Individual and combined effects of LVEF and obesity on sedentary behavior in patients with CAD (n = 200).

Variable	Category	Unadjusted			Adjusted		
		OR	95% CI	*P*	OR	95% CI	*P*
Model I	Individual influence						
	LVEF						
	Normal	1			1		
	Low	3.54	1.92–6.55	<.001	3.60	1.88–6.93	<.001
	Obesity						
	No	1			1		
	Yes	2.63	1.41–4.90	.002	3.56	1.75–7.26	<.001
Model II	Combined influence						
	Normal LVEF and without obesity	1			1		
	Low LVEF alone	4.52	1.39–14.77	.012	6.66	1.89–23.44	.003
	Obesity alone	3.91	1.84–8.31	<.001	4.11	1.87–9.02	<.001
	Low LVEF and with obesity	9.38	3.78–23.27	<.001	13.98	5.19–37.69	<.001

Adjusted for sex and age.

CAD = coronary artery disease, CI = confidence interval, LVEF = left ventricular ejection fraction, OR = odds ratio.

In the adjusted Model I, which examined the individual effect of LVEF or obesity on sedentary behavior, patients with low LVEF had a higher risk of sedentary behavior than those with normal LVEF (OR = 3.54, 95% CI = 1.92–6.55, *P <* .001). Similarly, patients with obesity had a higher risk of sedentary behavior than those without obesity (OR = 2.63, 95% CI = 1.41–4.90, *P =* .002).

Model II examined the combined effect of LVEF and obesity on sedentary behavior. After adjusting for confounding factors, patients with low LVEF and obesity had the highest risk of sedentary behavior compared to those without normal LVEF or obesity (OR = 13.98, 95% CI = 5.19–37.69, *P <* .001). Additionally, patients with low LVEF alone (OR = 6.66, 95% CI = 1.89–23.44, *P =* .001) or obesity alone (OR = 4.11, 95% CI = 1.87–9.02, *P <* .001) had a higher risk of sedentary behavior than those with normal LVEF and without obesity.

## 4. Discussion

This study investigated the combined effect of LVEF and obesity on sedentary behavior in patients with CAD. In this study, approximately 55.5% of the patients with CAD showed sedentary behavior for >8 h/d. This result is consistent with that of a previous study investigating sedentary behavior in older patients with hypertension using national data.^[25]^ Additionally, a cross-sectional study, which reported an average sitting time of 8 h/d in 278 participants in a heart rehabilitation program, also supports our study’s results.^[26]^ Overall, these studies suggest that sedentary behavior is highly prevalent among patients with CAD, with many patients spending long hours engaged in sedentary behavior. Therefore, reducing sedentary behavior and increasing physical activity are important for managing CAD and its associated health risks.

In this study, patients with CAD who engaged in sedentary behavior had a higher prevalence of low LVEF (78.4%) or obesity (43.2%) than those without sedentary behavior. This finding is consistent with those of previous studies that reported an association between low LVEF or obesity and CAD.^[18,27]^ Furthermore, a cross-sectional study supported this finding by demonstrating that individuals with lower levels of physical activity exhibit lower LVEF values than those who are more physically active.^[11]^ This suggests that the amount of time patients with CAD spend on sedentary behavior can vary according to the severity of their disease, including LVEF.^[11]^

Moreover, in this study, 34.2% (n = 38) of the patients with sedentary behavior had low LVEF and obesity, which were significantly higher than those without sedentary behavior (n = 11, 12.4%). This result aligns with previous studies showing that a low LVEF or obesity is associated with sedentary behavior in patients with cardiovascular diseases.^[12,19,28]^ Our results are similar to those of an intervention study, which reported that patients with CAD who are obese are less active and engage in more sedentary behavior than those with normal weight, both during and after cardiac rehabilitation.^[19]^ Patients with a low LVEF may be unable to engage in physical activities because of compromised cardiac function.^[29]^ Furthermore, engaging in sedentary behavior, which requires low levels of physical activity and prolonged sitting or reclining, can negatively affect cardiovascular health, particularly in individuals with a low LVEF.^[19]^ Therefore, determining the correlation between obesity and sedentary behavior is essential for implementing effective interventions and encouraging healthier lifestyles. Healthcare professionals should also prioritize early assessment of LVEF and obesity in patients with CAD who exhibit sedentary behavior. Additionally, future studies should focus on identifying LVEF and obesity as potential risk factors for recurrent events in patients with CAD and prolonged sedentary behavior.

Importantly, our study revealed a significant finding regarding the combined effects of low LVEF and obesity. Patients with CAD with low LVEF and obesity had a significantly higher risk of sedentary behavior, approximately 14.0 times higher than that of those without low LVEF or obesity. The combined effect of low LVEF and obesity on sedentary behavior was more significant than the individual effect of low LVEF or obesity alone. This finding highlights the importance of proactive management strategies focusing on improving daily physical activity and effectively controlling weight. Such management plays a crucial role in the secondary prevention and management of sedentary behavior in patients with CAD.^[26]^ Therefore, for patients with CAD, healthcare professionals need to consider a tailored cardiac rehabilitation program that addresses heart function improvement and weight control.^[19]^ Furthermore, it is important to note that our study did not explore the interrelationships among LVEF, obesity, and sedentary behavior in patients with CAD. Therefore, future longitudinal studies are warranted to investigate the causal or bidirectional relationships between LVEF, obesity, and sedentary behavior in this patient population. Moreover, further studies are also required to establish the correlation between the numerous causes of exercise intolerance and various echocardiographic parameters, including LVEF, that elucidate these factors. Such studies may provide valuable insights into the complex interactions between these factors and their impact on CAD outcomes.

This study has some limitations. First, the data used were cross-sectional and obtained through convenience sampling, which may limit the generalizability of the findings and restrict our ability to establish causality between LVEF, obesity, and sedentary behavior in patients with CAD. Second, this study analyzed Koreans based on the Korean Society for Obesity guidelines of ≥25 kg/m^2^ for obesity; therefore, this study’s results should be generalized and interpreted cautiously. Additionally, future research using longitudinal designs, representative sampling methods, and large-scale studies using the World Health Organization’s obesity standards (BMI ≥ 30 kg/m^2^)^[30]^ may provide more robust evidence. Lastly, sedentary behavior assessment in this study relied on self-reported measurements, which are subject to social desirability and recall bias. Therefore, objective and validated tools, in addition to self-reports, should be considered in future studies to provide a more comprehensive understanding of sedentary behavior in patients with CAD. Despite these limitations, this study provides valuable insights into the association among LVEF, obesity, and sedentary behavior in patients with CAD. However, further studies using longitudinal designs and various measurement approaches are needed to strengthen the evidence in this field.

## 5. Conclusions

This study provides compelling evidence that the combined effect of low LVEF and obesity significantly increases the risk of sedentary behavior in patients with CAD. These findings highlight the importance of simultaneously identifying low LVEF and obesity to effectively reduce the risk of sedentary behavior and mitigate adverse health outcomes in patients with CAD. Based on this study’s result, healthcare professionals should prioritize secondary prevention strategies aimed at encouraging patients with CAD to adopt a more active lifestyle. Additionally, even small changes, such as taking regular breaks from prolonged sitting, can make a difference. Healthcare professionals can contribute to the overall well-being and long-term prognosis of patients with CAD by addressing the combined effect of low LVEF and obesity on sedentary behavior. However, further studies are needed to explore targeted interventions and evaluate their effectiveness in reducing sedentary behavior and improving cardiovascular outcomes in this patient population.

## Acknowledgments

The authors would like to thank the patients who participated in this study.

## Author contributions

**Conceptualization:** Mi Hwa Won.

**Data curation:** Mi Hwa Won.

**Formal analysis:** JaeLan Shim.

**Funding acquisition:** Mi Hwa Won.

**Methodology:** Mi Hwa Won.

**Resources:** JaeLan Shim.

**Software:** JaeLan Shim.

**Supervision:** JaeLan Shim.

**Validation:** JaeLan Shim.

**Writing – review & editing:** JaeLan Shim.
